# Supplementary material for: Association between oral microbiome and five types of respiratory infections: a two-sample Mendelian randomization study in east Asian population
Source: Front Microbiol. 2024 Apr 10;15:1392473. doi: 10.3389/fmicb.2024.1392473 (PMC11039966; doi:10.3389/fmicb.2024.1392473)
Supplement: Supplementary file 1 [file Data_Sheet_1.zip › Supplementary Materials/MR plots of saliva/Pneumonia/s__Prevotella_nigrescens_mgs_3566/leave_one_out.pdf]

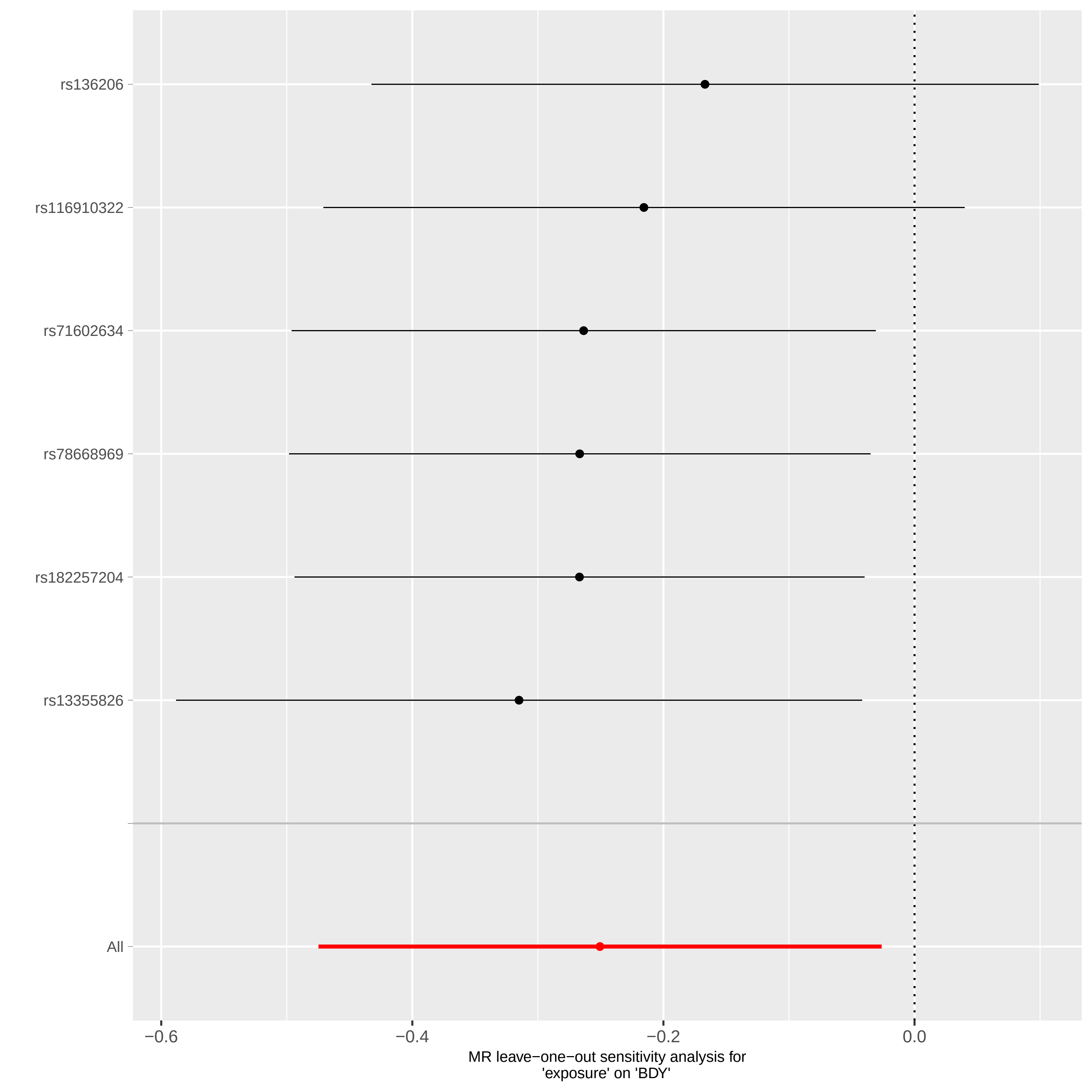
rs136206
rs116910322
rs71602634
rs78668969
rs182257204
rs13355826
All
−0.6
−0.4
−0.2
0.0
MR leave−one−out sensitivity analysis for
'exposure' on 'BDY'
